# Supplementary material for: Detection of large extracellular silver nanoparticle rings observed during mitosis using darkfield microscopy
Source: PLoS One. 2020 Dec 1;15(12):e0240268. doi: 10.1371/journal.pone.0240268 (PMC7707489; doi:10.1371/journal.pone.0240268)
Supplement: Attachment — Submitted filename: Rebuttle letter PLOS 1.docx [file pone.0240268.s001.docx]

**“Detection of Silver Nanoparticles within Cells and Large Extracellular Nanoparticle Rings using Darkfield Microscopy”**

Reviewers and editors comments

COMMENT: In this article the authors report detection of Ag nanoparticles of less that 100nm size using dark field microscopy.

The article stresses upon the importance of dark field imaging in detecting particles smaller than the detection limit. While this may be an advantageous technique in the field of nanoscience, the article fails to gather the audience in terms of any other interest and is a mismatch with the title. The article appears is verbose more as a laboratory report and less as an article contributing to the scientific knowledge.

The authors are suggested to modify the article title and rewrite the article, incorporating sufficient contribution in the field.

Response: The title was changed to focus on the large circles of nanoparticles that were observed following the mitosis of cells treated with nanoparticles. The new title is: “Detection of Silver Nanoparticles within Cells and Large Extracellular Nanoparticle Rings using Darkfield Microscopy”*.* The paper and figures have been rearranged and condensed. Two figures describing the technique have been put in the supplemental files section. Additional data on the measurements of the 45 the large circles of nanoparticles were added.

Reviewer #1: The article appears partially sound in terms of technicality as it fails to garner interest due to the writing style. It appears as a chapter of some thesis, with literature survey, dominant. There is not sufficient data to justify the title proposed.

Response: The paper has been extensively revised to focus on original data concerning the generation of a nanoparticle circles during the mitosis. The text was revised extensively to improve the writing style and, hopefully make it more interesting to the reader. The title was revised to reflect the new focus of the paper.

In addition, we would like to note, for the record, that the two senior authors (Zucker and Boyes) are well past writing their thesis.

Reviewer #2: The manuscript deals with the distribution of AgNPs in cellular organelles of epithelial cells (ARPE-19) studied by darkfield microscopy. Two different capping (PVP and citrate) for AgNPs are used, in a concentration range from 0.1 to 30 µg/ml. The novelty of this work lies on the accumulation of AgNPs in lysosomes and the formation of extracellular rings observed.
Although this findings can be of interest in the study of toxicity mechanisms of AgNPs in cells, the manuscript is not well discussed, there are not enough data that support some of the discussion (no images are included of AgNPs PVP for instance) and methodology has been already presented in a previous work (“Biophysical comparison of four silver nanoparticles coatings using microscopy, hyperspectral imaging and flow cytometry”, Plos One 14 (7) 2019) by the same authors, together with some of the discussion presented here. For all these reasons I do not consider this manuscript suitable for its publication in PLOS One.

Response: The novelty of this work does not lie on the accumulation of AgNPs in lysosomes but in the formation of extracellular rings observed during the mitosis. We have previously published on the AgNP uptake into lysosomes, and the extracellular rings were only mentioned briefly as a side issue. The organelle data are no longer a focus of the paper. The revised current submission now presents a more thorough evaluation of the rings, which we believe will be of interest to some readers. Some of the data on the uptake of AgNP into organelles has been placed in the supplemental files as it is useful to support some of the images showing extracellular rings with fluorescent cellular components with extra figures. The work in the paper focuses entirely on AgNP-citrate.

Reviewer #3: It is not clear from the piece of the work as to what is the message/finding that the authors would like to convey.
Whether it is detection of 80 nm AgNPs, or whether it is the observation of the decoration of nanoparticles around the cell organelles, or the formation of circular protein structures from agglomerated byproduct of cell division!
Even in this study, I did not find any cross-confirmation of the size of silver nanoparticles, nor there is any other evidence for the circular protein structure and this particular points is based on a hypothesis. Circular structures can form in case of any mixture of two immixible liquides and Ag nanoparticles will decorate the boundary, in any case.
Therefore, I am not sure how exactly your study would be able to significantly contribute in the advancement of the topic.
Probably the authors may need to gather more data and rework the manuscript.
I cannot recommend the publication.

Response: We agree that the previous version of the manuscript suffered from an unclear focus on several topics. In the resubmission, the major focus of the paper is now the formation of circular structures that attract agglomerated AgNP, we believe as a byproduct of cell division. These structures are not formed by immersible mixtures. We have never observed these formations in slides where the silver suspensions were added but no cells were present. Instead, they appear to be formed by positively charged cellular adhesion molecules located on the cell and slide interacting with negatively charged nanoparticles. This observation of extracellular circles is a unique finding that could be useful for scientists studying cell division. Additional data describing measurements on the size of 45 of these extracellular circles has now been added to the paper. The text has been reorganized and rewritten to focus on existence of these extracellular circles.

Regarding the issue of the physical/chemical characterization of the tested particles, we now have added supplemental materials to the manuscript provided from the NanoComposix company (SFig 9). Please note that this extensive characterization included transmission electron microscopy, dynamic light scatter, zeta-potential, UV-VIS, measurements of particle diameter and size distribution, surface area, chemical purity and endotoxin concentration. These data were reported specifically for the Lot number of materials used in the current experiments. Silver nanoparticles capped with the agents and provided in NanoComposix formulations have been reasonably stable in our hands, and are not prone to dissolution, degradation or transformation (Ortenzio et al, 2019). We therefore accepted this particle characterization as adequate for the purposes of the current studies.
