# Supplementary material for: Detection of large extracellular silver nanoparticle rings observed during mitosis using darkfield microscopy
Source: PLoS One. 2020 Dec 1;15(12):e0240268. doi: 10.1371/journal.pone.0240268 (PMC7707489; doi:10.1371/journal.pone.0240268)
Supplement: Attachment — Submitted filename: RESPONSE TO REVIEWER ARTICLE.odt [file pone.0240268.s002.odt]

Going through the abstract, the article and the conclusion, the authors have emphasized on the unusual appearance of silver nanoparticles around the cells undergoing mitosis. The authors leave the readers lost mentioning that these could be useful for future investigations. Perhaps the authors could emphasize more on this utility they foresee, thus, contributing to the scientific community. A histogram would be a desired add-on.
Response:  An explanation for how the nanoparticles rings originate during prophase is presented in the discussion and mentioned in the abstract.  A new histogram (Figure 5) of ring sizes has been added as suggested by the reviewer to summarize the sizes of rings observed.  An interpretation of the formation and thickness of the rings occurring during the prophase stage of mitosis has been proposed in the discussion.  Additional data on the size of the rings and their significance have been provided in the discussion.  The ring structure may be integrated with events that occur during prophase as the cell transitions between G2 restriction point and metaphase.  
The relationship of these ring structures appears to be another useful parameter to describe the events between prophase and metaphase which may correlate to the other events involving microtubules and chromosome condensation. 
The size of the nanoparticles in relation to the stage of the mitosis- can they be tabulated to give a rough idea to gauge the stage of cell division from the particle size, approximately
Response.  The nanoparticles were not observed to be different during the stage of the cell cycle or mitosis. Particles inside the cell are brighter and thus considered larger and clumped compared to particles outside the cell or in the rings.  The measurement of nanoparticles by darkfield microscopy was found to be about 0.37 μm.  The apparent size of the nanoparticles inside the cell are clumped and appear to be larger at 0.61 μm. These data are incorporated into the text of the manuscript.  It should be emphasized that dark field microscopy does not measure the actual size of the object or particle but shows the reflection of light from the location of the particle.  The darkfield observation of Ag nanoparticles show the particles to appear bigger than their actual physical size.  
The rings consist of particles that appear to have similar sizes to the extracellular particles distributed on the slide surface. It was not possible to measure the individual size of particles in the rings due to their high density, clumping and their location in a 3d structure.  

3. Perhaps, some more data to establish the technique could be provided. 
Response: A histogram of the rings structures was provided in in a new Figure 5. We also measured the size of the nanoparticles using Nikon elements by darkfield imaging. It was found that these particles have an apparent size of 0.37 μm by dark field microscopy, through measuring the area of reflected light as opposed to actual physical size of the particle which was described as 80 nm by the manufacturer.  The darkfield methodology can show particles below the diffraction limit of the microscope. The resulting image creates a spot that is larger than its physical size.  

4. Also, the authors could go through the manuscript once again and remove additional texts.
Response –The text has been revised and additional text removed.
