# Supplementary material for: Detection of large extracellular silver nanoparticle rings observed during mitosis using darkfield microscopy
Source: PLoS One. 2020 Dec 1;15(12):e0240268. doi: 10.1371/journal.pone.0240268 (PMC7707489; doi:10.1371/journal.pone.0240268)
Supplement: Attachment — Submitted filename: Response to reviewers.docx [file pone.0240268.s003.docx]

UNITED STATES ENVIRONMENTAL PROTECTION AGENCY

Center for Public Health and Environmental Assessment

Public Health and Integrated Toxicology Division

Research Triangle Park, NC 27711

OFFICE OF

RESEARCH AND DEVELOPMENT

**September 18, 2020**

Re: PONE-D-20-19210R1

Amitava Mukherjee, ME, Ph.D.

Academic Editor

PLOS ONE

Dear Dr Mukherjee,

Thank you and your peer reviewers for the careful evaluation of our manuscript entitled: “Detection of Large Extracellular Silver Nanoparticle Rings observed during Mitosis using Darkfield Microscopy”. For our previous revision, the reviewers indicated that the revised manuscript had addressed all the technical issues identified, but that the writing style and editorial quality were insufficient. The reviewers, however, did not identify any specific sections of the manuscript that needed attention.

Today, we are submitting a revised version of the manuscript which has been thoroughly revised to improve the editorial quality. Among other changes, we have removed redundancies, simplified and corrected sentence structures, corrected grammar, improved the consistency of acronyms and symbols, and corrected reference styles. We submit a “marked” version in which changes to the manuscript are indicated using the MS Word “track change” feature. You will see that the editorial revisions were extensive. We also submit a “clean” version in which all the changes were accepted. Overall, we believe, the quality and readability of the manuscript were improved, which should make it more interesting for the readers of PLOS ONE.

We hope that your reviewers will observe the improved quality of the manuscript and determine that it is now acceptable for publication in PLOS ONE.

Sincerely,

Robert Zucker, PhD

U.S. Environmental Protection Agency

Research Triangle Park, NC 27701

Email: [zucker.robert@epa.gov](mailto:zucker.robert@epa.gov)
